# Sequential tests for monitoring methods to detect elevated incidence – a simulation study

**DOI:** 10.1186/s12885-018-4259-z

**Published:** 2018-04-04

**Authors:** Tammo Konstantin Reinders, Joachim Kieschke, Antje Timmer, Verena Jürgens

**Affiliations:** 1 Leibniz Institute for Prevention Research and Epidemiology - BIPS, Achterstraße 30, Bremen, 28359 Germany; 2 Epidemiological Cancer Registry of Lower Saxony, Industriestraße 9, Oldenburg, 26121 Germany; 3 Carl von Ossietzky University of Oldenburg, Ammerländer Heerstraße 140, Oldenburg, 26111 Germany

**Keywords:** Cancer registry, Sequential test, Incidence, Cluster detection

## Abstract

**Background:**

Common cancer monitoring practice is seldom prospective and rather driven by public requests. This study aims to assess the performance of a recently developed prospective cancer monitoring method and the statistical tools used, in particular the sequential probability ratio test in regard to specificity, sensitivity, observation time and heterogeneity of size of the geographical unit.

**Methods:**

A simulation study based on a predefined selection of cancer types, geographical unit and time period was set up. Based on the population structure of Lower Saxony the mean number of cases of three diagnoses were randomly assigned to the geographical units during 2008–2012. A two-stage monitoring procedure was then executed considering the standardized incidence ratio and sequential probability ratio test. Scenarios were constructed differing by the simulation of clusters, significance level and test parameter indicating a risk to be elevated.

**Results:**

Performance strongly depended on the choice of the test parameter. If the expected numbers of cases were low, the significance level was not fully exhausted. Hence, the number of false positives was lower than the chosen significance level suggested, leading to a high specificity. Sensitivity increased with the expected number of cases and the amount of risk and decreased with the size of the geographical unit.

**Conclusions:**

The procedure showed some desirable properties and is ready to use for a few settings but demands adjustments for others. Future work might consider refinements of the geographical structure.

Inhomogeneous unit size could be addressed by a flexible choice of the test parameter related to the observation time.

## Background

Cancer monitoring and cluster detection have been and still are publicly debated at international level. The Center for Disease Control and Prevention (CDC) defines a cancer cluster *as a greater-than-expected number of cancer cases that occurs within a group of people in a geographic area over a period of time* [1]. In Germany, population-based cancer registries are responsible for further investigation in observed cancer clusters [2]. So far, active cancer monitoring is not common practice in the country. Recent cancer cluster detection practice is seldom prospective and rather initiated by requests by the public, physicians or health offices [3]. If a suspected elevated risk is reported, the corresponding spatial area will be explored. A common measurement is the standardized incidence ratio (SIR) which relates the cancer-specific cases in a region to the number of expected cases based on the rate in an appropriate reference population. A statistically significant elevation will be followed by further investigations to examine potential risk factor associations. However, this final step is known to be challenging due to several reasons such as complex disease etiology, long latency or human migration [3, 4].

The idea of an automatized search for spatially and temporally elevated cancer incidence has been debated in the past [5, 6]. While early detection is its main advantage pitfalls have also been discussed such as the high number of false positive results.

Classical testing methods predefine a fixed sample size in order to conclude on the parameter under consideration. In situations where data collection is time-consuming or costly, a more flexible approach which stops sampling as soon as a conclusion can be made may be more appropriate. Sequential tests provide this flexibility as they do not rely on a fixed sample size and stop as soon as a decision can be made which is checked after each iteration [7].

In 1999, a prospective three-stage monitoring for cluster detection was proposed [8]. During the first phase, the *search phase*, regions are preselected based on the consistency method (*Konstanzmethode*) [9]. For this approach, the observation time is divided into several periods. Ranks are assigned to the regions under study based on their cancer risk (indicated by a standardized rate or ratio). A final average rank is calculated for each region over the whole study period which serves as an indicator for areas under risk. In a next step, those areas are monitored prospectively. In this *observation phase*, a post-alarm-test is performed via the median- and mean-based technique proposed by Chen et al. [10]. During the final stage potential association with known risk factors is examined [8].

Lower Saxony is one of 16 German states located in the Northwest of the country covering more than 7.9 million of the German population (7,926,599 in 2015 [11]) and an area of around 47,614km^2^ divided in 762 municipalities. More than 47,800 cancer cases were reported in 2012 [12]. Initiated by an increased incidence of leukemia in a municipality, the Epidemiological Cancer Registry of Lower Saxony (EKN, Epidemiologisches Krebsregister Niedersachsen) was commissioned by the federal government to develop an automatized monitoring method which searches and identifies high cancer risk regions at small scale. Consequently, the investigations of 1999 were resumed by the EKN and the Governmental Institute of Public Health of Lower Saxony (NLGA, Niedersächsisches Landesgesundheitsamt), adjusted and further developed by considering sequential tests during the observation phase [13]. The adjusted approach starts with a search phase during which the SIR with confidence intervals for the last five years is calculated for all regional monitoring areas [8, 13]. Conspicuous areas will be incorporated into the second stage, the observation phase, which performs the sequential probability ratio test (SPRT). A yearly update of the test statistic will be provided and the area remains under surveillance until the SPRT reports a warning or all-clear signal [14].

In 2014, a pilot project was launched to validate the developed monitoring system applied for the cancer types acute myeloid leukemia (ICD-10 C92.0), renal cell carcinoma (ICD-10 C64) and mesothelioma (ICD-10 C45). These diseases were suggested by the EKN and NLGA as they are often discussed in relation with the environment and less biased in regard to data ascertainment. Furthermore, these diseases differ in their frequency ranging from a very rare (mesothelioma) to a relatively common diagnosis (renal cell carcinoma).

In this study, the monitoring method proposed by the EKN and NLGA, considering the SPRT as a statistical tool, was validated. In particular, we assessed the performance of the SPRT within a simulation study in regard to false-positive and -negative results, power, the observation time needed to receive a warning or all-clear signal by the SPRT and the influence of the size of the geographical unit on the reliability of the SPRT. Furthermore, we investigated the choice of (quantified) increased risk that should be identified by the test.

## Methods

### Monitoring method

The monitoring method is conducted in two stages, a search and an observation phase. At the start, all geographical units are under observation. During the search phase, the SIR and 95% confidence intervals are calculated for every geographical unit over a total time period of five years. Units with a significantly increased SIR are then selected for the observation phase while inconspicuous units get an all-clear signal and will no longer be observed.

During the observation, all remaining units undergo the SPRT introduced by Wald [15]. Its mathematical background was described in detail by Govindarajulu [16]. The SPRT is based on the likelihood ratio test. The basic idea is to evaluate the likelihood of an observation to be part of a certain underlying population. In this setting, we have to decide between two sets of hypotheses given an amount of acceptable uncertainty *α*. In order to add the sequential component, we do not only have to specify the significance level *α* but also the power 1−*β* of the test. Having set *α* and *β* we evaluate after every observation or at certain time points if the collected information suffices to draw a conclusion.

In the setting at hand, we choose *α*=*β*=0.05 and assume the number of cases of a geographical unit to follow a Poisson distribution $\mathbb {P}(\lambda)$ where *λ* denotes the expected number of cases. This leads to the following opposing hypotheses:

- the number of cases follow $\mathbb {P}(\lambda)$ vs.
- the number of cases follow $\mathbb {P}(r*\lambda)$,

where *r* is the increase in risk to be tested. The choice of *r* is crucial for the procedure performance. Every year the numbers of cases are evaluated with regard to the question whether a decision can be made based on the available information. If further information is needed, the unit remains under observation, otherwise the status will change and either a warning or an all-clear signal will be reported depending on the test result.

### Simulation study

For the monitoring at municipality level, we adapt an EKN-internal definition, the regional monitoring units (RMUs). They provide a mean RMU size of about 20,000 inhabitants and a minimum size of 5,000. Age group- and sex-specific numbers of cases for the three diagnoses mesothelioma (C45), renal cell carcinoma (C64) and acute myeloid leukemia (C92.0) and mean annual population numbers were provided by the Epidemiological Cancer Registry of Lower Saxony (see Table 1). Both datasets covered the years 2008 to 2012 and were given at RMU-level. Records with missing values or ambiguous sex status were excluded. The RMUs were classified regarding their population size (see Table 2).

**Table 1:** Total numbers of cases in Lower Saxony and expected numbers of cases at RMU level by diagnosis in the years 2008 to 2012

Diagnosis	Overall cases	Min	Median	Mean	Max
C45	958	0.13	0.31	0.49	12.63
C64	7129	0.93	2.34	3.68	93.97
C92.0	1761	0.23	0.58	0.91	23.21

**Table 2:** Classification of RMUs

Category	Number of	Total population	%
	RMUs	in category	
5100−10,000	101	818,900	10.30
10,000−30,000	236	3,692,100	46.60
30,000−100,000	43	1,873,900	23.60
100,000−522,600	8	1,545,600	19.50
5100−522,600	388	7,930,400	100

#### Scenarios

Three scenarios differing in the variation of the following parameters were applied (see Table 3):

1. *α*_*SPRT*_=*α* and *β*_*SPRT*_=*β* of the SPRT and the significance level *α*_*SIR*_ of the SIR,

**Table 3:** Parameter setting of scenario 1 to 21 distinguished by *α*_*SPRT*_, *β*_*SPRT*_, *α*_*SIR*_, the number of simulated RMUs under risk (RMU), the simulated risk for these selected RMUs (Risk) and the size of the simulated clusters (“Methods” section)

Scenario	*α*_*SPRT*_	*β*_*SPRT*_	*α*_*SIR*_	RMU	Risk	Method
1	0.05	0.10	0.05	–	–	–
2	0.05	0.05	0.05	–	–	–
3	0.05	0.01	0.05	–	–	–
4	0.01	0.10	0.05	–	–	–
5	0.01	0.05	0.05	–	–	–
6	0.01	0.01	0.05	–	–	–
7	0.05	0.10	0.01	–	–	–
8	0.05	0.05	0.01	–	–	–
9	0.05	0.01	0.01	–	–	–
10	0.01	0.10	0.01	–	–	–
11	0.01	0.05	0.01	–	–	–
12	0.01	0.01	0.01	–	–	–
13	0.05	0.05	0.05	10	1.50	20,000
14	0.05	0.05	0.05	10	2	20,000
15	0.05	0.05	0.05	10	4	20,000
16	0.05	0.05	0.05	10	1.50	40,000
17	0.05	0.05	0.05	10	2	40,000
18	0.05	0.05	0.05	10	4	40,000
19	0.01	0.05	0.05	10	2	20,000
20	0.05	0.10	0.05	10	2	20,000
21	0.05	0.05	0.01	10	2	20,000
2. fixed risk parameter of the SPRT, *r*=1.50 or *r*=2, or flexible *r*=*SIR*_*search*_,
3. the number of the RMU under risk (0 vs. 10) and the amount of the simulated risk (SR) increase (*SR*∈{1.50,2.00,4.00}).

Each scenario was simulated 1000 times. In every repetition, RMU under risk was randomly chosen and assigned a weight according to *SR*. Random allocation of cases to the RMU was done based on the RMU size multiplied with the assigned weight. Scenarios differing with regard to the risk parameter *r* were named M1.5, M2 and Msir, respectively.

The analysis starts with the simulation of five years, corresponding to the length of the search phase. SIR plus confidence interval are calculated and RMUs with significantly increased SIR according to the respective prespecified significance level will be selected for the observation phase. Prior to every observation phase, an additional year is simulated until SPRT terminated for all RMUs or, for computational reasons, until 100 years are reached. After each observation phase, the RMUs are assigned a status – red for warning, green for all-clear signal and yellow for remaining under observation.

The simulation study was conducted using the statistical software R version 2.15.1 [17].

## Results

For inspection of the results, all scenarios listed in the “Methods” section were considered. For reasons of clarity and compactness, only the most expressive results were selected for presentation in this paper. If an issue occurred or results were similar among all scenarios or methods, we decided to illustrate results by taking the example of the pilot project scenario and method.

### Specificity

In the search phase, the error rate fell short of the given significance level. For C45 (*α*_*SIR*_=0.05: 0.023, *α*_*SIR*_=0.01: 0.004) the lowest error rate was observed (C64: 0.036 and 0.007, C92.0: 0.026 and 0.004). In the observation phase, the given significance level was as well not exhausted. Thus, the percentage of false positives in total was by far below the expected percentage (e. g. with 35% of the expected number of false positives for *α*_*SIR*_=*α*=*β*=0.05, *r*=2 and no artificial clusters). This resulted in a very high specificity (greater than 0.99) for all scenarios. Small values of *α*_*SIR*_ and *α* led to even greater values for the specificity. The median number of false positives was 0 in every scenario.

For a significance level of *α*_*SIR*_=*α*=0.05, the amount of expected false positives was 0.05·0.05=0.0025=0.25*%* which lead to 0.0025·388=0.97 expected false positive alarms per year, since there were 388 RMUs. For C45 and without artificial clusters, 0.344 false alarms were produced in the simulation (in case of M2) which is 35% of the expected 0.97 false alarms. This would mean one false alarm every three years. In case of ten RMUs being under a risk of 2, one false alert would be expected in four years while a risk of 4 resulted in one expected false alarm in six to seven years.

Considering ten RMUs under a risk of *SR*=1.50, a number of 0.26 false positives were obtained (0.23 for *SR*=2). These decreased to 0.09 for a 4-fold increased risk, i. e. one wrong alarm within eleven years.

When simulating clusters, more RMUs were suspected during the search phase and hence underwent the SPRT. In the observation phase, the degree of exploitation decreased with an increasing *SR*. The real error rate was 0.01 (instead of 0.05) when *SR*=4. The number of false positives declined with a rising size of the simulated clusters leading to a higher specificity. This increase of specificity originated in the decreasing degree of exploitation of the designated significance level in the observation phase. The specificity was positively correlated with the choice of *α*.

### Sensitivity

For *SR*≤2 and diagnoses C45 and C92.0, sensitivity in the search phase reached a maximum of 0.50 whereas in the case of *SR*=4 sensitivity was observed to be greater than 0.83 (C45) and 0.94 (C92.0). For C64 a sensitivity of 0.61, 0.92 and greater than 0.99 was obtained for *SR*=1.50, *SR*=2 and *SR*=4, respectively. In the observation phase, M1.5 showed highest sensitivity greater than 0.88 (for most parameter choices considerably greater). In comparison with M1.5 and M2 results for Msir indicated worst performance with regard to sensitivity for C45 and C92.0 with values of 0.39 and 0.55, respectively, and *SR*=1.50. On the other hand, for C64 Msir showed higher sensitivity than M2.

Scenarios with assumed cluster size of 40,000 inhabitants were more sensitive than scenarios with cluster size of 20,000. Varying one parameter ceteris paribus in the search phase only *α*_*SIR*_=0.01 (vs. *α*_*SIR*_=0.05) differed in terms of sensitivity (smaller values). No major differences were detected during the observation phase.

### Observation time (years)

Overall, the individual as well as the maximum observation time decreased with increasing simulated risk. The average observation time did not noticeably depend on the simulated cluster size (20,000 vs. 40,000). Changing *α* from 0.05 to 0.01 led to longer observation times of single RMU while changing either *β* from 0.05 to 0.10 or *α*_*SIR*_ from 0.05 to 0.01 shortened observation time by a comparable amount.

For scenario *α*_*SIR*_=*α*=*β*=0.05 and *SR*=2, results indicated longer observation time for M1.5 compared to M2 and Msir for all three diagnoses (see Table 4).

**Table 4:** Average observation time (years) for a RMU, scenario 14

	M1.5	M2	Msir
C45	51.82	23.56	9.41
C64	8.72	3.85	5.90
C92.0	32.65	13.38	8.34

The maximum observation time was similar for all diseases considering Msir (C45: 21.38, C64: 23.37 and C92.0 21.67). Results for M1.5 and M2 were rather ambiguous. For M2 an observation time of 13.94 was reached for C64 while it took 66.06 years on average for all RMUs to terminate among the C45 diagnosis. A median of 100 years was estimated for M1.5 regarding C45 as well as C92.0 which is even underestimated since the observation time was limited (right-sided) to 100 years. For C64 an average time of 40.42 years was obtained.

### RMU size

**Fig. 1:**
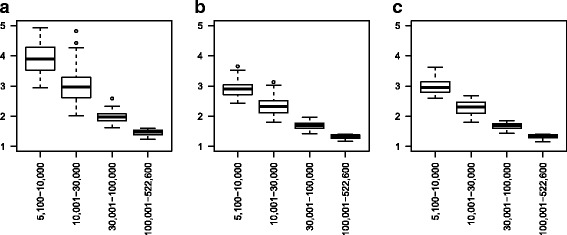
Boxplots of threshold values of the SIR by RMU size, *α*=0.05. **a** C45, **b** C64, **c** C92.0

Threshold values of the SIR, which is the level from which a RMU is considered suspicious and selected for the observation phase, were calculated. The threshold decreased with increasing RMU size and varied within each diagnosis ranging from 1.33–4.93, 1.08–2.08 and 1.16–3.62 for C45 (see Fig. 1a), C64 (see Fig. 1b) and C92.0 (see Fig. 1c), respectively.

The ranges of the corresponding critical number of cases, i. e. the number of cases needed for a RMU to be reported conspicuous, were 3–72 (mean = 6.05), 10–474 (mean = 26.06) and 4–135 (mean = 8.96) for C45, C64 and C92.0, respectively.

For fixed *r* the average observation time for a single RMU strongly depended on the RMU size. Results for the group of the smallest RMU provided observation times tenfold longer than the group of the largest RMU. For a flexible choice of *r*, only very small variation could be detected with average observation times between 8.80 and 10 years.

We found major differences in the detection of clusters regarding the RMU size. Large RMUs under risk are hardly detected. For example, 86% of clusters among C64 were detected, but only 0.01*%* in the group of RMUs with more than 100,000 inhabitants, while those with less than 10,000 inhabitants showed a detection rate of 0.97.

It is of major interest if an RMU under risk that becomes conspicuous due to its very large SIR can be confirmed in the observation phase. For Msir and C45, there were 837 cases with a maximum SIR of 9.9 of not confirmed RMUs having been conspicuous in the search phase. For M1.5 and M2, the corresponding numbers were 42 and 142, respectively. For Msir and C64, the maximum SIR was 6 (416 unconfirmed cases). M1.5 and M2 showed 122 and 589 unconfirmed cases. For C92.0 the corresponding values were between those of the other two diagnoses.

## Discussion

The aim of this study was to validate the performance of a recently developed and launched prospective monitoring method within a simulation study and to critically examine the performance of the SPRT. Based on the actual Lower Saxon population structure average numbers of cases of three cancer types were randomly assigned to the spatial units during 2008–2012. Scenarios which differed in regard to parameter choice of the increased risk (fixed vs. flexible) as well as the construction of artificial clusters were compared. Results suggest very high specificity for all methods and cancer types. Overall, diagnoses with a small number of cases resulted in considerably less false-positive reports. The results regarding sensitivity were rather ambiguous. Rare diseases produced much more false negatives than more frequent ones. Furthermore, results showed that heterogeneity of RMU size restricts reliability of the procedure regarding observation time (exclusive Msir) and sensitivity.

It was not the goal of this study to achieve significance but to test the monitoring procedure aiming to find a setup reliably providing alarm signals when an actual risk increase is present but simultaneously not producing false signals where no risk increase is given. This monitoring procedure is a case of massive multiple testing. Hence, it is very important to know its limitations and be accurate and cautious when it comes to interpretation of the results. The simulation showed a very high specificity. However, the number of false positives will increase by considering more diagnoses. Thus, an alarm should never be more than a starting signal for further investigation.

A high specificity is essential for prospective monitoring. Every false positive notification results in costs of both financial and non-financial nature, e. g. public attention. Secondly, future routine application of the method would consider numerous diagnoses under surveillance leading to an increasing risk of a randomly chosen RMU to be false positive. Hence, the high specificity is a benefit of the method. For any false positive notification, the method should ensure an adequate sensitivity. Therefore, a trade-off between specificity and sensitivity is needed. Low sensitivity was observed for low expected number of cases or slightly increased risk. A low sensitivity is not generally unacceptable. Lack of sensitivity can to a certain degree be absorbed by repeated search phases. Furthermore, even a method with a sensitivity of 0.99 could oversee existing clusters due to a low risk increase, time-limited clusters or clusters that cover only a small part of the population. On the other hand, a method with a sensitivity of 0.50 could be considered suitable if the number of false positives was low and the user was aware of its limitations.

In this study, the cancer cases were assumed to follow a Poisson distribution which is a common choice for count data. However, the assumption of the variance being the same as the mean might be violated in some scenarios, e. g. low number of cases. In this situation, other distributions such as the negative binomial or the zero-inflated equivalent may be more appropriate. The latter explicitly accounts for an excessive number of zero counts which might especially occur for diagnoses with low numbers of cases.

The inhomogeneity of the spatial units can to a certain extent be compensated by a flexible choice of the test parameter. The division of larger spatial units would be a potential solution for this problem. At the same time, joining the smallest units with their neighbors might improve observation times with a simultaneous expectable loss in sensitivity. Furthermore, a longer observation phase could lead to higher sensitivity and specificity.

Another option is the introduction of a “snooze” status. If there was no decision made for a specific RMU during the observation phase (in e. g. the first 3 observation periods), this RMU would “snooze” until it was again selected in one of the following search phases. The observation phase would then continue resulting in a longer time period for observation and a decision. An RMU could multiply receive a “snooze” status.

The choice of the test parameter is crucial for the performance of the monitoring procedure. Fixed and flexible choices were compared but there are alternatives to test in future work. The test parameter could be defined depending on the expected number of cases of the respective RMU, e. g. as the minimum SIR with which the RMU would get conspicuous during the search phase. Such an approach would require either a lower limit for the test parameter to avoid testing on non-relevant risk increases or the division of large RMUs to avoid such small critical values.

Numerous cluster detection approaches exist such as the spatial scan statistic [18], Moran’s I [19] or Cuzick-Edwards’ k-nearest neighbors [20]. Common practice in cancer cluster detection is rather retrospective and reactive responding to inquiries from the public [21]. In 1990, Rothman criticized this approach [6]. He raised several concerns about cluster studies. Among others, he mentioned the bias introduced by the already (by knowledge) influenced population prior to the cluster investigation and migration. A prospective monitoring design overcomes this drawback. We do not step into the trap of the Texan Sharpshooter’s Fallacy as the spatial units are defined beforehand. The study is based on confirmed cancer registry cases. Remaining concerns are that clusters may often be too small to allow for a controlled epidemiological study and that definitions of disease may be too vague.

Cancer usually has a long latency, meaning the time between exposure and disease manifestation. This may lead to incorrect residential assignments as the person might have moved during that period. Furthermore, cancer registry data include case-related information but lack information about environment-related risks.

This simulation study is based on the population structure in Lower Saxony of 2008 through 2012 and does not consider demographic change which may result in long observation periods.

## Conclusions

A final appraisal of the monitoring method is challenging and can’t be achieved. However, it can be concluded that its performance is mainly driven by the expected number of cases, meaning the larger the number of cases the higher the sensitivity and the shorter the observation time. Also, lower risk increase can be detected more easily which can be seen as another advantageous consequence.

A specificity above 0.99, sensitivity above 0.80, observation time below 10 years and the ability to detect RMUs with twofold increased risk would represent reasonable demands of the monitoring procedure. This would be feasible for diseases with similar or higher numbers of cases than renal cell carcinoma (C64). Diseases such as mesothelioma (C45) and acute myeloid leukemia (C92.0) would not fulfill these demands. This does not mean that the procedure is not applicable for C45 and C92.0 in general. Again, it is a matter of the demands.

## Abbreviations

EKN: Epidemiological cancer registry of lower saxony (Epidemiologisches Krebsregister Niedersachsen)
ICD: International classification of disease
NLGA: Governmental Institute of Public Health of Lower Saxony (Niedersä
chsisches Landesgesundheitsamt); RMU: Regional monitoring unit
SIR: Standardized incidence ratio
SPRT: Sequential probability ratio test
SR: Simulated risk
